# *FONPS6*, a Nonribosomal Peptide Synthetase, Plays a Crucial Role in Achieving the Full Virulence Potential of the Vascular Wilt Pathogen *Fusarium oxysporum* f. sp. *Niveum*

**DOI:** 10.3390/life15020142

**Published:** 2025-01-21

**Authors:** Jiaqi Li, Yanyang Gao, Bowen Li, Li Zhang, Yi Fang, Hongtao Zou, Xuhong Ye

**Affiliations:** 1 College of Land and Environment, Shenyang Agricultural University, Shenyang 110866, China; lijiaqi2023@syau.edu.cn (J.L.); gaoyanyang@163.com (Y.G.); libowenen@163.com (B.L.); 15390649309@163.com (L.Z.); 13658424354@163.com (Y.F.); zht@syau.edu.cn (H.Z.); 2 Key Laboratory of Arable Land Conservation (Northeast China), Ministry of Agriculture and Rural Affairs, Shenyang 110866, China; 3 National Engineering Research Center for Efficient Utilization of Soil and Fertilizer Resources, Shenyang 110866, China

**Keywords:** nonribosomal peptide synthetase, targeted disruption, complementation, allelochemicals, virulence, *Fusarium oxysporum*

## Abstract

*NPS6* is one of the nonribosomal peptide synthetase (*NRPS*) family members. The roles of *NPS6* in ascomycetes are well known, but its roles in *Fusarium oxysporum* are unidentified. We investigated its function in the growth, morphology, stress sensitivity, allelochemical secretion, and pathogenesis in *F. oxysporum* (*FoNPS6*). The partial deletion of *FoNPS6* orthologs (Δ*FON-NPS6*) resulted in hypersensitivity to H_2_O_2_ and KO_2_, iron depletion, and reduced virulence. Full virulence was restored by complementation. Δ*FON-NPS6* not only inhibited spore formation but also displayed hyphal growth patterns that differed significantly from the wild-type strain. Plant leaching released allelochemicals, which *FON-NPS6* broke down. All of these findings show that *FoNPS6* quantitatively increases *F. oxysporum*’s pathogenicity.

## 1. Introduction

*Fusarium oxysporum*, a globally distributed soil-borne pathogen, is notorious for causing a variety of wilt diseases in economically significant crops. This filamentous fungus is highly diverse, with specific forms (forma specialis) adapting to infect a broad spectrum of host plants, including the well-known Fusarium wilt in watermelon, caused by *Fusarium oxysporum* f. sp. *Niveum* [1]. The pathogenicity of *F. oxysporum* is largely attributed to its ability to produce a wide range of bioactive secondary metabolites, which are crucial for the successful colonization and infection of its host plants. A key component in the pathogenic arsenal of *F. oxysporum* and other related species is the production of nonribosomal peptides (*NRPs*), which are synthesized by nonribosomal peptide synthetases (*NRPSs*) [2]. These enzymes play a critical role in the biosynthesis of many virulence factors, including phytotoxins that promote disease progression.

*NRPSs* are a group of enzymes characterized by their intricate structure, primarily enabling the production of small peptides without relying on ribosomal machinery for protein synthesis. Fungal *NRPS*-derived compounds are critical in mediating plant–microbe interactions. Research has established that *NRPS* metabolites produced by phytopathogenic ascomycetes function as phytotoxins. These toxins, specifically AM toxin and HC toxin synthesized by the apple pathotype of *Alternaria alternata*, are crucial contributors to the pathogenicity of the fungus. Both AM and HC toxins exhibit high toxicity not only to the host organisms but also to the producing fungi themselves [1,2,3]. A total of 12 genes encoding *NRPS* were identified in the *Cochliobolus heterostrophus* genome. *NPS6* was found to be virulent only to maize when only a single deletion was made [2]. In numerous instances, various pathogen species have adopted shared pathogenic strategies, leading to a phenomenon where pathogenic gene function is maintained through vertical inheritance and exposure to common host defense selection factors during pathogenesis in the same or related hosts. *NPS6* has been confirmed to be conserved across filamentous ascomycetes, similar to observations in *C.heterostrophus*. The disruption of *NPS6* orthologs in *Cochliobolus miyabeanus*, a pathogen of rice; *Fusarium graminearum*, which infects wheat, maize, and barley; and *Alternaria brassicicola*, a pathogen affecting cruciferous plants led to reduced virulence toward its specific hosts as well as heightened susceptibility to reactive oxygen species and iron-deficient conditions. *NPS6* was demonstrated to be responsible for extracellular siderophore biosynthesis [3].

*Achromobactin*, a siderophore produced by *Erwinia chrysanthemi*, has been shown to contribute to the pathogen’s virulence [4]. *Glycophilic bacteria* produced by the closely related apple disease *Erwinia amylovora* causes a successful infection with desferrioxamine, which is mandatory for apple owners [5]. In contrast, siderophiles do not appear to be necessary for virulence in the crown gall pathogen Agrobacterium tumefaciens [6] or another closely related species, *Erwinia carotovora subsp carotovora* [7]. This suggests that *siderophores* have distinct roles in plant–microbe interactions in various pathosystems. *NPS6* has been identified as a conserved factor essential for the virulence of the plant pathogen *Fusarium graminearum* [8], and as there is not yet a report about the *NPS6* gene in *Fusarium oxysporum*, its function should be further determined.

Phytochemicals can be released into the environment through various processes, such as plant leaching, root exudation, volatilization, and the decomposition of plant residues [9,10]. Current research indicates that allelochemicals, such as phenolic acid (PA), can modify cell membrane permeability and inhibit the function of H+-ATPase enzymes, which subsequently causes damage to both DNA and proteins. In more severe scenarios, this disruption can stimulate the formation of lipid peroxidation signaling molecules. The cumulative impact of these alterations can result in extensive cellular injury, potentially compromising cellular integrity and, in extreme cases, inducing cell death. This sequence of events highlights the complex biochemical interactions by which allelochemicals exert their toxic effects on cellular structures and functions [11]. Another adverse effect of allelochemicals on plants is that they tend to alter the abundance and community structure of *rhizobial flora*, disrupting the balance of the microbial community [12,13]. Since *NPS6* has been confirmed to have a relation to the virulence of *F. graminearum* [14], we speculate that *NPS6* might affect the *allelochemical* secretion of *F. graminearum*. However, whether *NPS6* has the same role in plants has not been determined before.

Therefore, we aim to identify the *NPS6* gene in *F. oxysporum*, clarify the role of the *NPS6* gene in the pathogenic behavior of *F. oxysporum*, and then verify the effects of *NPS6* in *F. oxysporum* on allelochemical secretion in plants.

## 2. Materials and Methods

### 2.1. Fungal Isolates, Plant Materials, and Growth Conditions

*F. oxysporum* f. sp. *niveum* race 1 was obtained from Jiangsu Academy of Agricultural Sciences; it was isolated from watermelon which had been infected with *Fusarium* wilt disease and was stored at −80 °C with 50% glycerol as a microconidial suspension. Genomic DNA was extracted from the *Fusarium oxysporum* strain using the E.Z.N.A.™ Fungus DNA Kit (Omega Bio-Tek, Norcross, GA, USA) following the manufacturer’s instructions for fungal DNA isolation. PCR amplification was performed using Phusion High-Fidelity DNA Polymerase (New England Biolabs, Ipswich, MA, USA). The pathotype of the isolate was confirmed periodically by plant infection assays. Unless mentioned otherwise, it was grown on CM at 24 °C under continuous fluorescent light. The minimal medium (MM) was complete medium (CM) without yeast extract, acid-hydrolyzed casein, and enzymically hydrolyzed casein.

Soils collected from Nanjing, Jiangsu Province, China, were sterilized by triple autoclaving (121 °C for 1 h over three consecutive days) with the following chemical properties: organic matter content: 51.23 mg/g; total nitrogen: 3.13 mg/g; total phosphorus: 2.97 mg/g; pH: 6.8. Watermelon seeds (Sumi No. 5) were purchased from the Jiangsu Academy of Agricultural Sciences, China. This cultivar has no resistance to *Fusarium* wilt.

### 2.2. PCR Cloning of the FONPS6 Gene

Genomic DNA from the *F. oxysporum* strain was isolated using the E.Z.N.A.™ Fungus DNA Kit (Omega Bio-Tek, Norcross, GA, USA) in accordance with the manufacturer’s protocol. PCR amplification was carried out using Phusion DNA polymerase (NEB) following the provided instructions. Using the specified primer pair, orthologs of the *F. oxysporum FONPS6* genes were successfully amplified via PCR from the *NPS6* genes of *F. graminearum* and *A. brassicicola*. This approach enables the comparative analysis of gene function and conservation across these different fungal species, providing insights into the role of *NPS6* orthologs in pathogenicity and potential evolutionary relationships among the phytopathogens (NPF: 5′-ATGGGAGACGTTCAGTCGTC-3′; NPR: 5′-CTAGATCAAATCGTTAGGGTT-3′). PCR products of approximately 6kb were inserted into the pGEM-T Easy vector (Promega, Madison, WI, USA) for sequencing.

### 2.3. The Targeted Disruption of the FONPS6 Gene

Following two rounds of PCR amplification, a chimeric fragment was generated, consisting of a portion of the *FONPS6* gene fused with the HYG segment [15] using a Phusion DNA polymerase (NEB). This process facilitated the construction of a recombinant DNA segment essential for further analysis and experimentation. The HYG gene is responsible for encoding a phosphatase that confers resistance to chlorothalonil. This gene operates under the regulatory influence of the trpC promoter and terminator derived from Aspergill us niger. In the first round, HYG fragment was amplified from pUCATPH [16] with the primers M13R and M13F, respectively. The 5′*NPS6* fragment was amplified by PCR with the primers FP1(5′-GCGTTATGACGTACCTGTTCTTCG-3′) and M13 RRP1(5′-TCCTGTGTGAAATTGTTATCCGCTCTCGCCACCGATACCAAGACT-3′), and the 3′*NPS6* fragment was amplified with M13 FFP2 (5′-GTCGTGACTGGGAAAACCCTGGCGCAGATTGGTATTGAGGCTGACGAG-3′) and RP2 (5′-CTCGTCAGCCTCAATACCAATCTG-3′) from the genomic DNA of *F. oxysporum*. In this study, the sequences highlighted in primers M13RP1 and M13FFP2 represent oligonucleotides that are completely complementary to the sequences of M13R and M13F primers. During the second PCR round, the *5′NPS6::HYG* disruption fragment was created by directly fusing the 5′*NPS6* and HYG segments without the need for additional primers. Subsequently, the HYG fragment was combined with the *3′NPS6* segment to produce the *HYG::3′NPS6* disruption fragment. The resulting fragments, each at a concentration of 10 µg/mL, were then mixed and directly introduced into the protoplasts of the wild-type strain using CaCl_2_ and polyethylene glycol [17]. Transformants were selected on CM agar supplemented with 200 µg/mL hygromycin (Roche Applied Science, Penzberg, Germany) and screened for successful integration. The deletion of *FONPS6* in the transformants was verified through PCR using M13R and M13F primers. Transformants with positive PCR results for the remaining three primers were used as controls, while the PCR results for M13R and M13F were negative.

### 2.4. Complementation

The *F. oxysporum FONPS6* ORF (~5 kb) with 5′ and 3′ flanking sequences (each ~2 kb) was PCR-amplified with the primer pair FP1/RP2. Approximately 5 µg of the PCR product and 10 µg of plasmid pII99, containing the nptII gene [18,19], were used to transform strain Δ*FON-NPS6*. Secondary screening of the transformants was performed on CM without salts [20] containing 400 µg/mL of G418. G418-resistant transformants were then selected and tested for hygromycin B sensitivity. Through a polymerase chain reaction (PCR) analysis, it was confirmed that the *FONPS6* fragment successfully substituted the hygB gene within the 418R hygBS transformant.

### 2.5. Characterization of Growth

To evaluate the growth characteristics of the mycelium, 8 mm diameter plugs were cut from the periphery of fungal colonies of both the WT and Δ*FON-NPS6* strains on CM agar. These plugs were subsequently positioned at the center of two distinct media: CM and MM. After incubating the cultures for 72 h at 25 °C in light, the diameters were measured. The data were collected from two independent experiments, each comprising three replicates. Morphology of strains was analyzed with scanning electron microscopy. The germination of asexual spores (conidia) was evaluated by preparing a suspension of the spores from both the WT strain and Δ*FON-NPS6*, cultured on CM, at a spore suspension concentration of 105/mL. Subsequently, 300 µL of the suspension was transferred into 1.5 mL test tubes and incubated at 30 °C for 1, 2, or 3 h. A drop of the spore suspension was then placed onto a well of a glass slide, and the samples were observed under a bright-field microscope. The number of germinated spores was counted for each sample, with a total of 50 spores being counted per sample.

### 2.6. Stress Sensitivity Assays

Cultivation of the WT and Δ*FON-NPS6* strains was carried out on solid minimal medium including stressors as well as without them. An 8 mm mycelial plug, taken from the edge of fungal colonies grown on MM agar that does not contain FeSO_4_ (the sole iron source in MM, rendering it iron-deficient), was placed in the center of the medium. To assess the susceptibility of the *FON-NPS6* strain, three or four independent transformants were tested under each condition, with triplicate experiments being carried out for each. All stress assay chemicals were obtained from Sigma. Two oxidants, H_2_O_2_ (3%) and the superoxide generator KO_2_, were used at final concentrations of 20 mM and 40 mM, respectively. Iron-depleted conditions were induced by using the iron chelator 2DP at a final concentration of 400 µM. Additionally, WT and Δ*FON-NPS6* strains were grown on MM, iron-deficient MM, and MM supplemented with 1 mM ferric citrate, ferrous ammonium sulfate, or ammonium ferric sulfate for 3 days. Five replicates per strain were prepared for each condition, and three independent Δ*FON-NPS6* strains were analyzed. Colony diameters were measured, and data were evaluated using one-way ANOVA.

### 2.7. HPLC Analysis of Phenolic Acids

The *rhizobial* chamber was divided into a central zone (30 mm wide) and left and right zones (30 mm wide) separated by a 0.22 m *acetocellulose* membrane, with the central zone being the side with a rough surface intended to prevent the movement of roots and microorganisms to the other side. In each zone of the *rhizobial* chamber, a piece of nylon cloth was placed 1 mm from the membrane, and in three spaces in the zone, including the space between the film and the first nylon cloth, pre-prepared sterilized soil was placed, and these samples between the membrane and the nylon cloth were used for the adsorption of exudates migrating from the central zone through the membrane as the watermelon root system was kept within the central zone.

Plants thinned to four were sprayed with a spore suspension (7.2 × 10^5^ CFUs/g) of dry soil inoculated with WT or *FON-NPS6* strains. Plants were ensured to be grown under greenhouse conditions with temperatures ranging from 20 to 30 degrees Celsius during the day and 20 to 25 degrees Celsius at night. Forty-five days after sowing into *rhizobial* boxes, the soil between the film and nylon cloth was collected and brought back to air dry.

Allelochemicals were extracted following the method outlined by Martens [21]. The extracts were analyzed with an Agilent 1200 semipreparative HPLC system (Santa Clara, CA, USA). Phenolic acids were separated using a synthetic solvent gradient elution method under certain assay conditions. The gradient program was designed as follows: 0–27 min, 100–90% solvent A, 0–10% solvent B; 27–42 min, 90–85% solvent A, 10–15% solvent B; 42–50 min, 85–70% solvent A, 15–30% solvent B; 50–60 min, 70–50% solvent A, 30–50% solvent B; 60–70 min, 50–0% solvent A, 50–100% solvent B. Standards for 4-hydroxybenzoic acid, vanillic acid, ferulic acid, benzoic acid, 3-phenylpropanoic acid, and cinnamic acid were sourced from Sigma-Aldrich (Steinem, Germany), and stock solutions were created in methanol. The HPLC chromatogram for PAs was created following the previously outlined method with an injection volume of 5 μL.

### 2.8. Virulence Assays

To assess the virulence of WT, Δ*FON-NPS6*, and complementary strains, spores were resuspended in sterile distilled water supplemented with 0.02% (*v*/*v*) Tween 20, with a final spore concentration of 1 × 10^7^ CFU/mL. Three-week-old watermelon plants (ten per assay) were transplanted into pots with a volume of 640 cm^3^. The plants were placed in a mist chamber for 24 h, followed by incubation in a growth chamber with a 16 h light/8 h dark cycle at 24 °C. Virulence was assessed 5 days post-inoculation using the method outlined by Pritsch et al. [22]. *Fusarium* wilt symptoms in each plant were assessed using a 0–5 scale as follows: 0 means the plant remained entirely healthy; 1 means less than 10% of the leaves showed wilting; 2 means 11–20% of the leaves wilted; 3 means 21–50% of the leaves wilted; and 4 means 50–100% of the leaves were affected.

The disease index was calculated using the following formula:

Disease Index = Σ (level value × plant number)/(total plant number × 4) × 100%

Leaf point inoculation was conducted following a modified version of the method by Rahman et al. [23]. In brief, 5 μL droplets of spore suspension (10^5^/mL) in water were applied to the leaves of 4-week-old watermelon plants (five plants per assay).

Control plants received the same volume of water without fungal spores. Inoculation was followed by a 7-day incubation period in a growth chamber. Symptoms were visually assessed. Additionally, each virulence assay was conducted a minimum of three times for every pathosystem. Watermelon roots were placed in Erlenmeyer flasks filled with a microspore suspension at a concentration of 5 × 10^6^ µg/mL. The flasks were then incubated in a growth chamber at a speed of 80 rpm for 24 h to evaluate the roots’ penetration capabilities. After incubation, the samples were examined using scanning electron microscopy and analyzed following the methodology outlined by Sanderson et al. [24].

## 3. Results

### 3.1. Mutagenesis of FoNPS6, a Gene Encoding Nonribosomal Peptide Synthetase 6 in F. oxysporum

The *NPS6* gene (*FoNPS6*) was cloned from *F. oxysporum* f. sp. *niveum* race 1(WT). The gene contains a 6272 bp open reading frame (ORF). A BLAST search against the complete genome database of *F. oxysporum* using the amino acid sequence of *F. graminearum NPS6* identified a single potential ortholog, FOXG_09785.2, which encodes a hypothetical protein consisting of 2053 amino acids. The predicted *FoNPS6* protein has a conserved domain architecture including a phosphopantetheine attachment site, a condensation domain, and an amino acid adenylation domain. The predicted protein exhibited a high degree of identity to previously characterized fungal *NPS6* (e.g., *F. graminearum*, 91%; *Gibberella zeae* PH-1, 81%; and *Aspergillus brassicicola*, 76%). *NPS6* has been identified to contribute to the virulence of *F. graminearum* [25]. Given the significant sequence conservation between *F. oxysporum* and *F. graminearum*, as indicated by sequence homology, this gene plays a crucial role. Therefore, a further investigation into the function of *FoNPS6* in *F. oxysporum* was conducted.

### 3.2. Deletion of FoNPS6 Affected Multiple Growth-Related Characteristics

**Figure 1:**
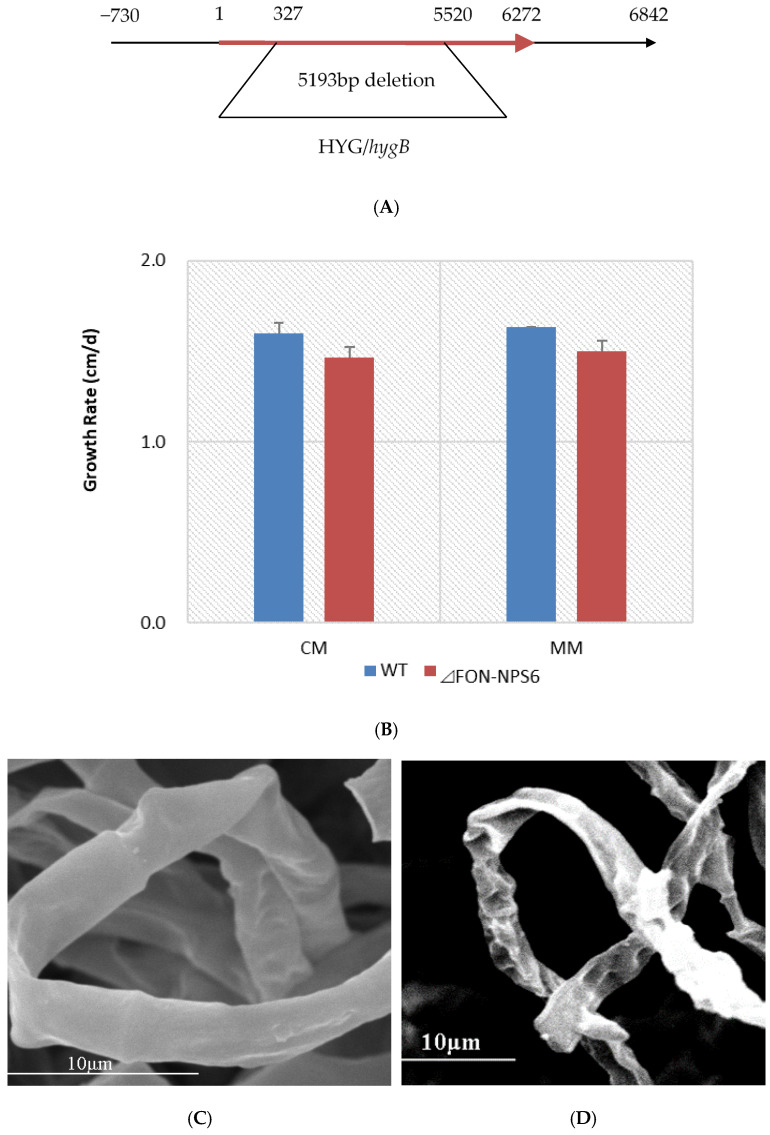
Deletion of *FoNPS6* affected multiple growth-related characteristics. (**A**) *FoNPS6* was deleted with split marker constructs. Binding sites for primers used to confirm mutants are noted with asterisk. (**B**) Growth rate of average colony diameters of WT and Δ*FON-NPS6* strains grown on MM or CM. Error bars indicate 95% confidence intervals. Statistically significant difference (*p* < 0.05) in growth was observed. (**C**,**D**) Scanning electron microscopy analysis of morphology of WT (**C**) and Δ*FON-NPS6* (**D**) strains grown on MM. Surface of WT mycelium was smoother than that of Δ*FON-NPS6* mycelium.

The role of *FONPS6* in *F. oxysporum* infection was investigated by altering the 5193 bp *FONPS6* genomic sequence (*FON-NPS6*), and finally, a partial *FONPS6* knockout was obtained (Figure 1A). Growth characteristics of Δ*FON-NPS6* on two different solid media, including complete medium (CM) [25] and minimal medium (MM) were compared with WT strains. Δ*FON-NPS6* strains showed reduction in radial growth relative to these WT strains (Figure 1B) on MM and CM. The surface topography of the hyphae from the WT and *FON-NPS6* strains was visualized using scanning electron microscopy, which showed that the surface of the WT mycelium was visibly smoother than the surface of the *FON-NPS6* mycelium (Figure 1C,D). Conidia of WT and *FON-NPS6* strains were taken out and inoculated into liquid MM at different times for microscopic examination. Differences in shoot elongation, septum formation, and mycelial meristem were found between the two strains.

The germination rate of asexual spores of the WT strain was lower than that of the *FON-NPS6* strain during the first 2 h, but the difference was not significant during the last 1 h (Table 1). These results demonstrate that *FoNPS6* did not play an essential role in plant growth and conidiation in culture.

### 3.3. Deletion of FoNPS6 Leads to Hypersensitivity to Superoxide and H_2_O_2_ and to Iron Depletion

**Figure 2:**
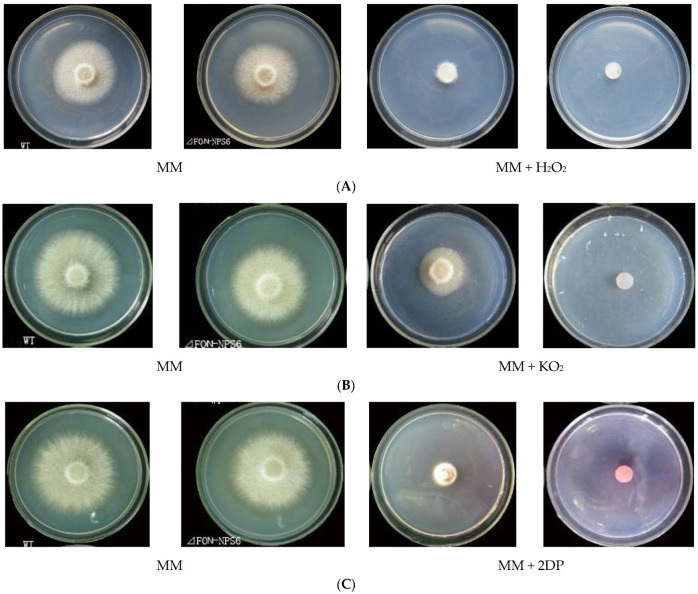

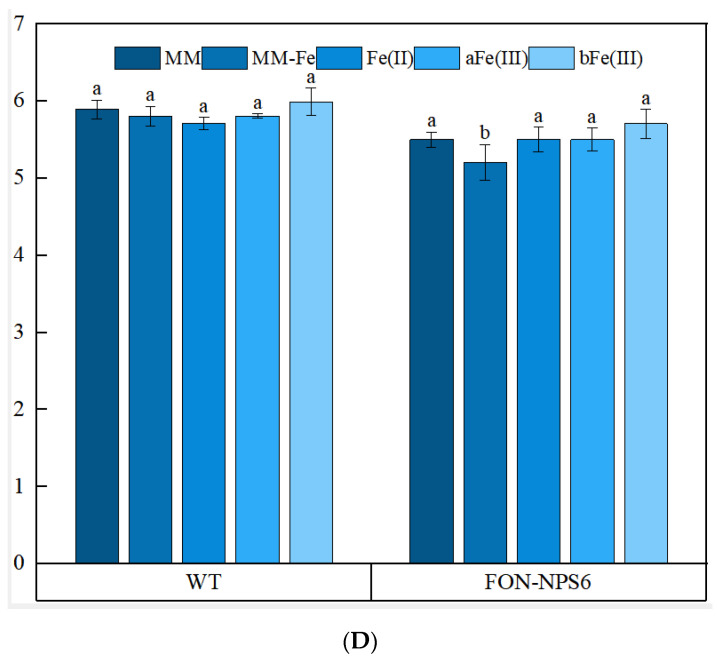
The deletion of *FoNPS6* leads to hypersensitivity to superoxide and H_2_O_2_ and to iron depletion. Different lowercase letters above the bars indicate significant differences between treatments (*p* ≤ 0.05). (**A**) The hypersensitivity of the Δ*FON-NPS6* strain to 20 mM of H_2_O_2_. Three-day-old cultures of the WT (**top row**) and Δ*FON-NPS6* (**bottom row**) strains. The growth of the Δ*FON-NPS6* strain was completely inhibited on the MM, and the WT grew under the same conditions. (**B**) The hypersensitivity of the Δ*FON-NPS6* strain to 40 mM of KO_2_. Three-day-old cultures of the WT (**top row**) and Δ*FON-NPS6* (**bottom row**) strains. The growth of the Δ*FON-NPS6* strain was completely inhibited on the MM, and the WT grew under the same conditions. (**C**) The hypersensitivity of the Δ*FON-NPS6* strain to 400 μM of 2DP (iron chelator). Three-day-old cultures of the WT (**top row**) and Δ*FON-NPS6* (**bottom row**) strains. The growth of the Δ*FON-NPS6* strain was completely inhibited on the MM, and the WT grew under the same conditions. (**D**) The hypersensitivity of the Δ*FON-NPS6* strain to iron deficiency (MM without FeSO_4_). The error bars indicate 95% confidence intervals. A statistically significant difference in size (*p* < 0.05) was observed. No differences in sensitivity to iron addition (1 mM of ferrous ammonium sulfate (Fe(Ⅱ)) or ammonium ferric sulfate (aFe(Ⅲ)) or ferric citrate (bFe(Ⅲ)) were detected between the WT and Δ*FON-NPS6* strains. Different letters on the columns layer represent significant differences (*p* < 0.05).

To further investigate the function of *FoNPS6*, we evaluated the sensitivity of *FOC-NPS6* strains to various stress conditions, including oxidative stress induced by superoxide and H_2_O_2_, as well as iron translocation and supplementation. The *FOC-NPS6* strain exhibited heightened sensitivity to oxidative stress triggered by 40 mM KO2 and 20 mM H_2_O_2_ (Figure 2A,B), and also to iron deficiency, induced by the iron chelator 2,2′-dipyridyl (2DP) and MM lacking FeSO_4_, the sole iron source (Figure 2C,D). No differences in sensitivity to 1.0 mM of iron addition were detected between the WT and Δ*FON-NPS6* strains regardless of whether ferric citrate, ferrous ammonium sulfate, or ammonium ferric sulfate was used (Figure 2D).

### 3.4. PA Degradation by ΔFON-NPS6

**Figure 3:**
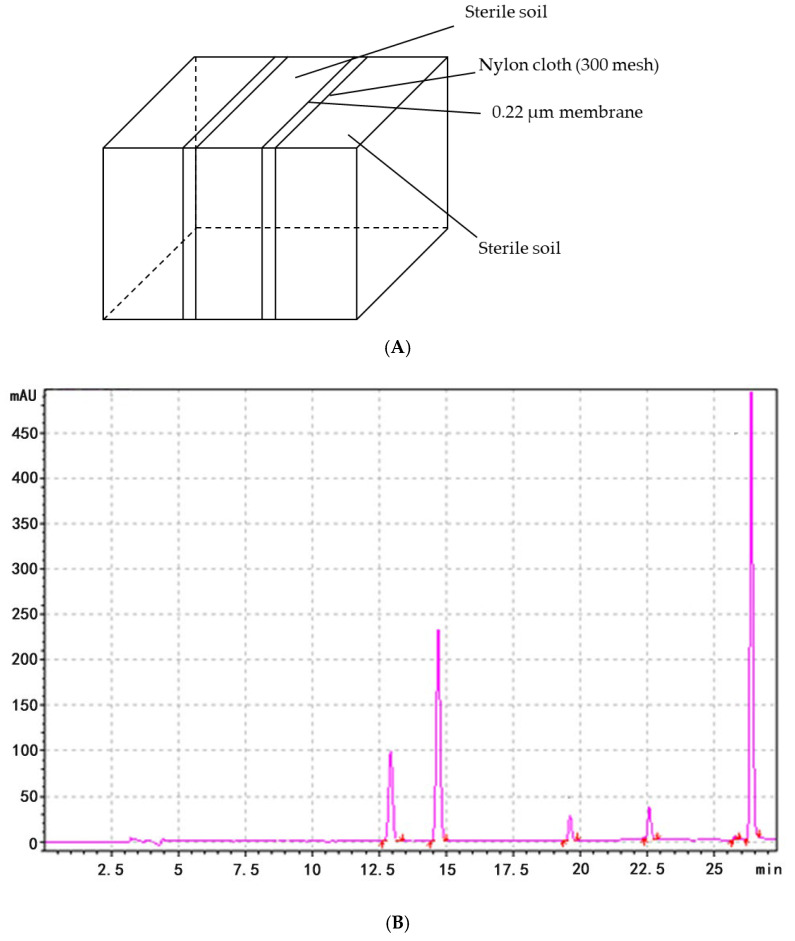

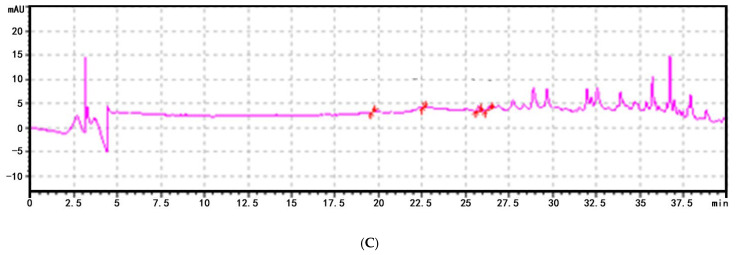
*FONPS6* affected PA secretion of plants. (**A**) Illustration of *Rhizobox*. (**B**) Chromatogram of HPLC of extraction from soil of cucumber in FOC strains. (**C**) Chromatogram of HPLC of extraction from soil of cucumber in Δ*FON-NPS6* strains.

To assess whether *FoNPS6* influenced the secretion of phenolic acids (PAs) by watermelon plants, a custom-made *Rhizobox* was employed to collect root exudates from the soil (Figure 3A). In the soil surrounding the wild-type (WT) plants, six specific PAs—4-hydroxybenzoic acid, vanillic acid, ferulic acid, benzoic acid, 3-phenylpropanoic acid, and cinnamic acid—were detected (Figure 3B). However, in the soil around the Δ*FON-NPS6* mutants, only ferulic acid, benzoic acid, acrylic acid, and cinnamic acid were identified, with concentrations significantly lower than those found in the WT. Specifically, secretion levels were reduced by 66.2%, 99.8%, 80.4%, and 99.9%, respectively. Notably, hydroxybenzoic acid and vanillic acid were completely absent in the mutant exudates (Figure 3C). An analysis of the HPLC chromatograms showed that both the number and intensity of peaks in the exudates from the Δ*FON-NPS6* treatment were markedly reduced compared to those from the WT treatment.

### 3.5. Mutant ΔFON-NPS6 Lacking FoNPS6 Gene Reduced Virulence, and Complementation Restored Full Virulence

**Figure 4:**
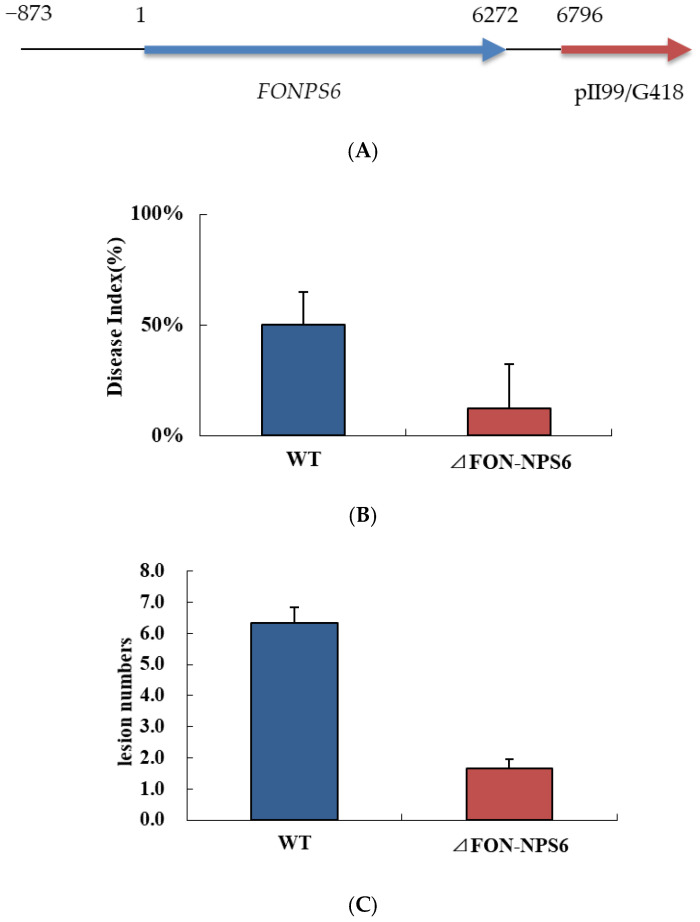

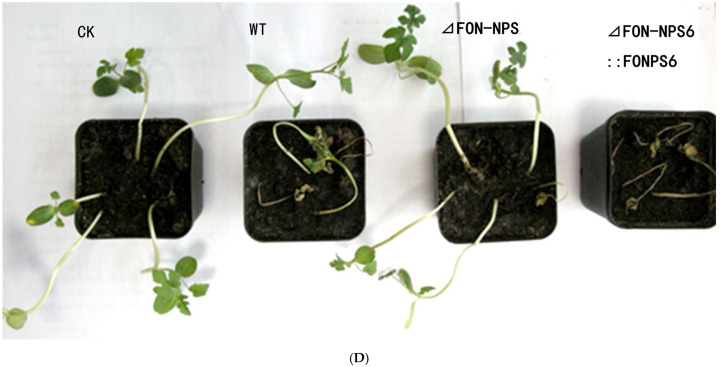
Δ*FON-NPS6* mutant lacking *FoNPS6* gene reduced virulence. (**A**) Δ*FON-NPS6* was complemented with split marker constructs. (**B**) Disease indices of watermelon inoculated with WT and Δ*FON-NPS6* conidia (7.2 × 10^5^ CFU per gram of dry soil). Error bars indicate 95% confidence intervals. Statistically significant difference in size (*p* < 0.05) was observed. (**C**) Average vertical lengths of lesions on watermelon leaves formed by WT and Δ*FOC-NPS6* strains. Error bars indicate 95% confidence intervals. Statistically significant difference in size (*p* < 0.05) was observed. (**D**) Disease symptoms of plants grown using soil mix in plate are shown. Plants were inoculated with water (first), WT (second), Δ*FON-NPS6* (third), and Δ*FON-NPS6*:: *FONPS6* (fourth).

To determine whether *FoNPS6* inactivation impacted pathogenicity, virulence assays were quantified on watermelon plants inoculated with the WT, the mutant, and the complemented mutants (Δ*FON-NPS6::FONPS6*) (Figure 4A) in a separate experiment. After 30 days of watermelon growth, the WT treatment exhibited a *Fusarium* wilt disease index of 92%, while the Δ*FON-NPS6+FONPS6* treatment showed an index of 90%. In contrast, the Δ*FON-NPS6* treatment resulted in a significantly lower index of only 12.5% (Figure 4B). The mean vertical lengths of lesions induced by the WT strain and the Δ*FON-NPS6* and Δ*FON-NPS6::FONPS6* treatments demonstrated that the WT and Δ*FON-NPS6::FONPS6* treatment lesion size were much longer (Figure 4C). So, the Δ*FON-NPS6-*complemented mutants had a restored full-disease phenotype that was not significantly different in disease progression from inoculations with the fully virulent WT. Disease progression was also significantly higher in the complement mutants and WT than in the *FONPS6* knockout mutants (Figure 4D).

**Figure 5:**
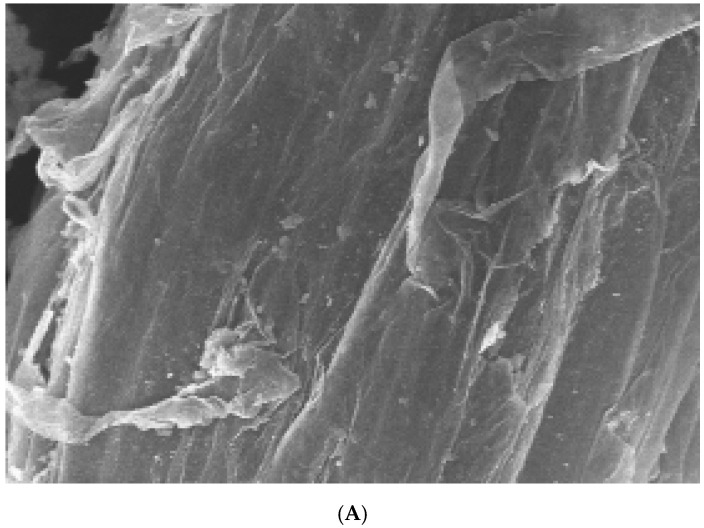

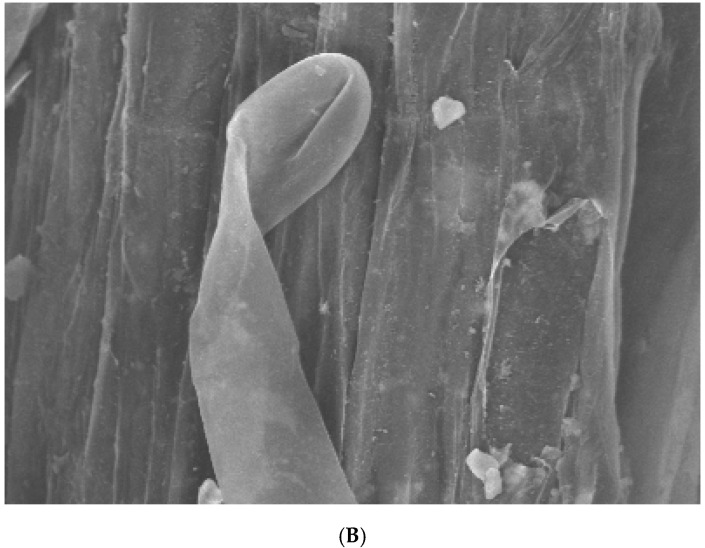
(**A**,**B**) *NPS6* contributes to penetration of watermelon roots. Scanning electron microscopy analysis of watermelon roots 24 h after inoculation with microconidia of *F. oxysporum* f. sp. *niveum* WT (**A**) or Δ*FON-NPS6* (**B**) strain. (**A**) shows fungal hyphae entering root through openings at intercellular junctions. Arrows point to penetration events. (**B**) shows multiple hyphal fusion events indicated by Δ*FON-NPS6*.

To investigate penetration by *F. oxysporum* into the surface of watermelon roots, scanning electron microscopy was used to analyze the roots inoculated with microconidia from both the WT and Δ*FON-NPS6* strains. The results show the hyphae penetrating the root via gaps at the intersections of the epidermal cells (Figure 5A,B). In the WT, the frequency of penetration was higher than that in the Δ*FON-NPS6* strain.

## 4. Discussion

The *NPS6* gene encodes a product recognized as an extracellular glycoconjugate, which acts as a conserved virulence factor in pathogenic ascomycetes, including the distantly related cereal pathogen *F. graminearum* [25]. However, although *F. oxysporum* has a close genetic relationship with *F. graminearum*, it does not belong to the ascomycete species. Therefore, whether this gene in *F. oxysporum* has the same or different role with those in *F. graminearum* and other ascomycetes species was studied in this study.

Here, we identified the *F. oxysporum NPS6* gene by its homology to the *NPS6* protein from *F. graminearum*, *G. zeae* PH-1, and *A. brassicicola*. These three proteins exhibit a high degree of amino acid sequence similarity and possess a shared domain architecture, including a phosphopantetheine attachment site, a condensation domain, and an amino acid adenylation domain. The presence of these three highly conserved genes in different species is a phenomenon that suggests either a common origin that may be recent or an evolutionarily conserved function that limits genetic diversity [26].

To dissect the function of *FoNPS6* in *F. oxysporum*, we generated an independent *F. oxysporum* strain in which *FONPS6* had been deleted (Δ*FON-NPS6*). Compared to the WT strain, the *FON-NPS6* strain was observed to have a common characteristic phenotype similar to head growth failure and hypersensitivity to low iron. The Δ*FON-NPS6* strain exhibits increased sensitivity to conditions of low iron availability, and its strong iron-chelating ability led us to hypothesize that *FoNPS6* plays a role in plant colonization by providing the essential nutrient iron to the pathogen. Further characterization revealed that the Δ*FON-NPS6* strain promotes the germination of asexual spores at first but is normal at last. These results indicate that *FoNPS6* just affects the rate of germination of asexual spores and not the number [27].

The mutant of Xanthomonas oryzae pv. oryzae, known as the fur (ferric uptake regulator) variant, which exhibited an overproduction of extracellular siderophores, also demonstrated reduced virulence in rice and heightened sensitivity to H_2_O_2_ [28,29]. It has also been reported that the deletion of *NPS6* reduces the pathogenicity of heterotrophic molds and *Fusarium graminearum* on maize and renders maize insensitive to H_2_O_2_ [30,31]. We found that *FoN-NPS6* exhibits hypersensitivity to H_2_O_2_ and the superoxide generator KO_2_. Thus, the role of *FoNPS6* in this characteristic function is similar to its role in *Fusarium graminearum*, as described above.

Δ*FON-NPS6* significantly reduced virulence on watermelon. Furthermore, to test that the reduced levels of virulence in Δ*FON-NPS6* was due to deletion of *FoNPS6*, Δ*FON-NPS6* was complemented, and complementation restored the WT disease phenotype. Root colonization in hydroponic cultures was observed using the same pathogenic and non-pathogenic strains previously employed in the study by Voß et al. [32]. In the study, the pathogenic strain appeared to be a better and faster colonizer than the biocontrol strain. In our study, evidence suggests that the interaction of *F. oxysporum* with solid surfaces elicits a swift reaction, which requires *FoNPS6*. This may be one of the reasons why the *FoNPS6* gene is associated with enhanced virulence. However, further studies are needed to draw conclusions about the role of *NPS6*-mediated iron metabolism in pathogenicity.

Overall, our findings are consistent with previous reports on *F. graminearum* [33], suggesting that *NPS6* plays a widely conserved role in fungal pathogenicity. However, there is limited information regarding the mechanisms by which the *NPS6* mutant degrades phenolic acids (PAs). This study demonstrated that in cultures inoculated with Δ*FON-NPS6*, the concentration of PAs was significantly lower compared to those inoculated with the wild-type strain. Allelochemicals, especially water-soluble, low-molecular-weight PAs, are known to exhibit fungitoxic effects against various fungi [34]. These compounds can have toxic effects on fungal species, either synergistically or even in a superimposed manner, resulting in a decrease in the microbial population in the area surrounding these toxins [35]. Notably, cinnamic acid and 4-hydroxybenzoic acid have been shown to enhance the growth status and infection probability of *F. oxysporum* f. sp. *cucumerinum* [36], whereas vanillin and 4-hydroxybenzoic acid promote *Fusarium oxysporum* infection in peanut plants [37]. Therefore, the observed decrease in the *allelochemical* content in soils treated with *FON-NPS6* is likely due to the degradation of PAs by this strain. Additionally, pathogens with greater infective capacity tend to elicit a reduced secretion of plant allelochemicals. Nevertheless, for the optimal application of Δ*FON-NPS6*, further research is needed to investigate the processes that occur after allelochemicals are transported into the cells of Δ*FON-NPS6* in future studies.

## 5. Conclusions

The partial deletion of *FoNPS6* orthologs led to a heightened sensitivity to oxidative stressors as well as to conditions of iron depletion. Δ*FON-NPS6* could impair spore formation and exhibited hyphal growth patterns that markedly diverged from those of the wild-type strain. Interestingly, plants release allelochemicals through leaching, which *FoNPS6* possesses the ability to degrade. Therefore, *FoNPS6* quantitatively increases *F. oxysporum* pathogenicity.

## 6. Institutions

- The College of Land and Environment, Shenyang Agriculture University, Shenyang, Liaoning, China
- The National Engineering Research Center for Efficient Utilization of Soil and Fertilizer Resources, Shenyang, Liaoning, China
- The Northeast Key laboratory of conservation and improvement of cultivated land (Shenyang), Ministry of Agriculture and Rural Affairs, P.R., China

## Figures and Tables

**Table 1:** Percent *F. oxysporum* spore germination in vitro ^a^.

Test	Hrs After Inoculation	WT	Δ*FON-NPS6*
1	1	6% (3/50)	8% (4/50)
	2	48% (24/50)	54% (27/50)
	3	82% (41/50)	80% (40/50)
2	1	0% (0/50)	2% (1/50)
	2	42% (21/50)	58% (29/50)
	3	84% (42/50)	84% (42/50)
3	1	0% (0/50)	6% (3/50)
	2	36% (18/50)	48% (24/50)
	3	78% (39/50)	92% (46/50)
Average	1	2%	5.3%
	2	42%	53.5%
	3	81.3%	85.3%

^a^ A total of 50 spores were counted for each strain and time point.

## Data Availability

The data is unavailable due to privacy or ethical restrictions.
